# Encapsulation Strategies for Natural Bioactives in Clean-Label Meat Preservation: A Review

**DOI:** 10.3390/foods15132407

**Published:** 2026-07-07

**Authors:** Guliz Haskaraca, Hatice Sıçramaz

**Affiliations:** Food Engineering Department, Engineering Faculty, Sakarya University, Sakarya 54050, Turkey; haticesicramaz@sakarya.edu.tr

**Keywords:** encapsulation, natural bioactives, essential oils, meat preservation, clean-label, nanoemulsions, controlled release, antimicrobial activity

## Abstract

The increasing demand for clean-label meat products has accelerated interest in natural bioactive compounds, including essential oils, plant polyphenols, and bacteriocins, as alternatives to synthetic preservatives. These compounds have the potential to enhance product safety and shelf life while meeting consumer expectations. Many natural bioactives exhibit antioxidant and antimicrobial activities, enabling them to reduce lipid oxidation and inhibit the growth of spoilage and pathogenic microorganisms in meat systems. Despite these benefits, their practical application remains limited by instability, volatility, poor solubility, and undesirable sensory effects. Encapsulation technologies have emerged as effective approaches to overcome these limitations by enhancing stability, controlling release behavior, and improving compatibility with complex meat matrices. This review synthesizes evidence from 154 studies published between 2010 and 2026 on the application of encapsulation technologies, including microencapsulation, nanoemulsions, liposomes, and cyclodextrin-based systems, for natural bioactives in meat systems. Encapsulated bioactive delivery systems are evaluated by integrating spoilage mechanisms, delivery system design, and application strategies. Encapsulation approaches are discussed in terms of structure–function relationships, release behavior, and interactions with meat components. Application strategies, including direct incorporation, edible coatings, and active packaging, are comparatively analyzed based on their functional performance in meat systems. Overall, nanoscale delivery systems are particularly effective in improving the dispersion, stability, and functional performance of hydrophobic bioactives, while controlled-release systems offer prolonged protection but often exhibit reduced predictability when translated from model systems to real meat matrices. Current challenges related to scalability, cost, regulatory constraints, sensory impact, industrial implementation, and the safe design of sustained-release antimicrobial systems are also addressed, thereby providing a framework for the rational development and implementation of effective clean-label preservation strategies in meat systems.

## 1. Introduction

The increasing demand for minimally processed foods with consumer-recognized ingredients has led to a shift toward clean-label preservation strategies in meat and meat products [1]. Although there is no universally accepted regulatory definition, the term “clean label” is commonly used to describe products formulated with ingredients perceived by consumers as natural, familiar, and minimally processed. In this context, encapsulation technologies have emerged as promising tools for enhancing the stability, controlled release, and functional performance of natural bioactive compounds, thereby supporting the development of clean-label preservation strategies in meat systems. Conventional preservatives, including synthetic antioxidants and antimicrobials, remain effective; however, growing consumer concerns regarding their safety and long-term health implications have accelerated the shift toward natural alternatives [2].

Natural bioactive compounds, including plant-derived compounds such as essential oils and phenolics, as well as bioactive peptides and bacteriocins of microbial or protein-derived origin, have attracted considerable attention due to their antimicrobial and antioxidant properties [3]. Despite these advantages, their practical application in meat systems remains highly challenging, primarily due to chemical instability, volatility, limited water solubility, and strong sensory impact [4]. In addition, interactions with meat components, including proteins and lipids, may further reduce their local availability and functional performance within the meat matrix [5].

These limitations highlight a fundamental gap between the intrinsic functionality of natural bioactives and the complex physicochemical environment of meat systems. As a result, direct incorporation strategies often fail to achieve consistent and effective preservation outcomes under real processing and storage conditions [6]. Encapsulation technologies have therefore emerged as a promising approach to enhance stability, control release behavior, and improve compatibility with food matrices.

Despite extensive research on individual encapsulation techniques, a system-level understanding of how encapsulation design parameters (e.g., carrier material, particle size, and structural organization) influence functional performance within complex meat matrices remains limited [7]. Furthermore, comparative insights into when and why specific encapsulation systems should be preferred are still insufficient, particularly in relation to their release behavior, matrix interactions, scalability, cost-effectiveness, and industrial applicability. Although several review articles have discussed natural preservatives or encapsulation approaches in meat systems, most have focused on specific bioactive compounds, preservation effects, or individual encapsulation techniques separately.

This review aims to critically evaluate the role of encapsulated natural bioactive compounds as a promising strategy for clean-label-oriented preservation in meat systems, with a focus on delivery technologies, matrix interactions, release behavior, and the transition from laboratory-scale research to practical applications. Although the available literature has expanded significantly, current studies remain largely fragmented, often focusing on individual encapsulation systems or specific bioactive compounds without fully considering their performance within complex meat matrices [8]. Several reviews have previously addressed topics related to natural bioactives and encapsulation for meat preservation. Ojeda-Piedra et al. [4] focused specifically on nano-encapsulated essential oils as preservation systems for meat and meat products, whereas Gómez et al. [9] reviewed the microencapsulation of antioxidant compounds and their application in meat processing. Domínguez et al. [10] provided a broader overview of plant-derived bioactive compounds, including their extraction, characterization, encapsulation, and potential use in meat products. However, these reviews primarily focused on specific bioactive classes, individual encapsulation approaches, or broader food applications. Unlike previous reviews, the present review comparatively examines different encapsulation strategies from a structure–function perspective while also emphasizing release mechanisms, practical applicability, and challenges associated with industrial implementation. Therefore, a more structured and integrative critical evaluation that links encapsulation design, release behavior, and application strategies in meat systems is needed, contributing to a clearer understanding of their potential for effective and scalable implementation.

## 2. Literature Search and Review Methodology

This review was designed as a structured narrative review with a critical synthesis approach rather than a full systematic review, and no formal review protocol was pre-registered. Major scientific databases, including Web of Science, Scopus, and PubMed, were searched for articles published between 2010 and 2026. This period was selected to focus on recent advances in encapsulation technologies and their applications in clean-label meat preservation, particularly developments related to nano-delivery systems, active packaging, and controlled release approaches. Earlier foundational studies were also considered when they provided important conceptual or methodological background. The search strategy combined keywords such as “encapsulation”, “microencapsulation”, “nanoemulsion”, “natural bioactives”, “essential oils”, “meat preservation”, “antioxidant”, and “antimicrobial”. The keywords were combined using Boolean operators (AND, OR) to maximize retrieval of relevant studies. A representative search string used for database searches was: (“encapsulation” OR “microencapsulation” OR “nanoemulsion” OR “liposome”) AND (“natural bioactives” OR “essential oils” OR phenolics OR bacteriocins) AND (“meat” OR “meat products” OR “meat preservation”) AND (“antioxidant” OR “antimicrobial”). Searches were conducted within the title, abstract, and keyword fields, with minor adaptations according to the requirements of each database. The final literature search was conducted in May 2026. A total of 558 records were initially identified from the selected databases. After removal of 103 duplicate records, 455 records remained for title and abstract screening. Following the exclusion of 247 records during the screening stage, 208 reports were sought for retrieval. Of these, 12 reports could not be retrieved, resulting in 196 full-text articles being assessed for eligibility. After full-text evaluation, studies that were not directly related to meat systems, lacked encapsulation approaches, or provided insufficient methodological relevance were excluded. Ultimately, 154 studies were included in the qualitative synthesis.

Studies were included based on the following criteria: (i) investigation of natural bioactive compounds, (ii) application of encapsulation or delivery systems, and (iii) evaluation within meat or meat products. Studies focusing exclusively on in vitro systems, conference abstracts, and studies lacking sufficient relevance to meat systems were excluded unless they provided important mechanistic or technological insights relevant to food applications. The selected studies were critically analyzed with respect to encapsulation strategy, structural properties, release behavior, matrix interactions, and functional performance in meat systems. Particular emphasis was placed on identifying relationships between system design and practical applicability, including scalability, cost-effectiveness, and industrial relevance in meat systems. Although this review was not designed as a full systematic review, the literature selection process was transparently summarized using a PRISMA 2020-style flow diagram and is presented in Supplementary Figure S1.

## 3. Spoilage Mechanisms in Meat Systems and Preservation Strategies

**Figure 1:**
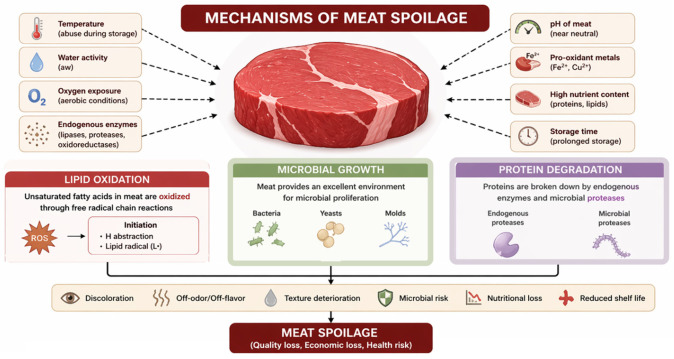
Schematic representation of the main mechanisms of meat spoilage.

Meat and meat products are highly perishable foods in which quality deterioration results from a combination of oxidative, microbiological, and physicochemical processes occurring simultaneously during storage. These degradation pathways not only limit shelf life but also directly affect consumer acceptance through undesirable changes in flavor, color, texture, and safety [4,11]. A schematic overview of the major spoilage mechanisms occurring in meat systems is presented in Figure 1.

Lipid oxidation is one of the primary mechanisms responsible for quality deterioration in meat systems. It leads to the formation of hydroperoxides and secondary oxidation products such as aldehydes and ketones, which contribute to rancidity, off-odors, and reduced sensory quality [12]. In addition, pigment oxidation, particularly the conversion of bright red oxymyoglobin to brown metmyoglobin in red meat, results in discoloration and reduced consumer acceptability. Lipid oxidation and pigment oxidation are closely interconnected and may collectively accelerate quality deterioration during storage. These oxidative processes are further promoted by environmental factors such as oxygen exposure, light, and temperature [13].

Microbial spoilage represents another major pathway of deterioration, driven by the growth and metabolic activity of spoilage microorganisms and, in some cases, pathogenic bacteria that compromise product safety. Microbial metabolism can lead to protein degradation, production of volatile compounds, biofilm formation, and accumulation of metabolites associated with off-odors and reduced product safety. The extent of microbial spoilage is strongly influenced by storage conditions, packaging systems, and processing practices, making effective preservation essential [14]. In addition to oxidative and microbial deterioration, physicochemical changes such as protein denaturation, moisture loss, and structural modifications also contribute to quality degradation by affecting texture, water-holding capacity, and overall sensory quality. These changes often occur simultaneously with lipid oxidation and microbial growth, accelerating product deterioration during storage [12].

The relative importance of spoilage pathways varies considerably depending on product type, packaging conditions, and storage temperature. In fresh red meat stored under aerobic conditions, lipid oxidation and myoglobin oxidation are major factors affecting sensory quality, whereas vacuum-packaged products are more frequently limited by the growth of lactic acid bacteria and other anaerobic or facultative anaerobic microorganisms [15,16]. In modified-atmosphere packaging systems, the dominant spoilage mechanisms depend on gas composition and storage conditions, although microbial growth generally remains a major determinant of shelf life [17]. In processed meat products with higher fat contents, oxidative deterioration often becomes more pronounced, while temperature abuse can accelerate both microbial growth and oxidative reactions across product categories [18]. These differences highlight the need for preservation strategies and encapsulation systems tailored to specific product characteristics, storage conditions, and target spoilage pathways.

Conventional preservation strategies have traditionally relied on synthetic antioxidants and antimicrobial agents to control these deterioration pathways. However, the widespread use of synthetic preservatives is increasingly challenged by consumer concerns regarding artificial additives, the demand for shorter and more recognizable ingredient lists, and potential regulatory or labeling restrictions. In addition, some conventional preservation systems may not adequately meet current expectations for naturalness, sustainability, and minimal processing. Therefore, increasing consumer demand for clean-label products has shifted attention toward natural bioactive compounds, including plant-derived extracts, essential oils, and peptides [19]. These compounds exert their preservative effects through multiple mechanisms, including free radical scavenging, metal chelation, membrane disruption, and enzyme inhibition, thereby targeting both oxidative and microbial spoilage pathways [14,20].

Despite their functional potential, the practical application of natural bioactives in meat systems is often constrained by limitations related to stability, solubility, and sensory impact. These challenges are further amplified by matrix-specific factors, including diffusion barriers, phase partitioning, and interactions with proteins and lipids, which can restrict the accessibility, distribution, and functional activity of bioactive compounds [14]. As a result, maintaining effective concentration at target sites throughout storage remains challenging, highlighting the need for delivery systems capable of improving bioactive protection, distribution, and controlled release within complex meat matrices.

In this context, encapsulation technologies offer a promising strategy to bridge the gap between their functional potential and practical application, as discussed in detail in subsequent sections.

## 4. Clean-Label Strategies in Meat Preservation

In recent years, increasing consumer awareness regarding food safety, ingredient transparency, and health concerns has driven a strong shift toward clean-label products in the meat industry. Consumers increasingly prefer meat products formulated with natural ingredients and produced using minimal processing, while avoiding synthetic additives such as artificial antioxidants and preservatives. This trend has encouraged the development of alternative preservation strategies capable of maintaining product quality and safety while aligning with consumer expectations [20].

**Figure 2:**
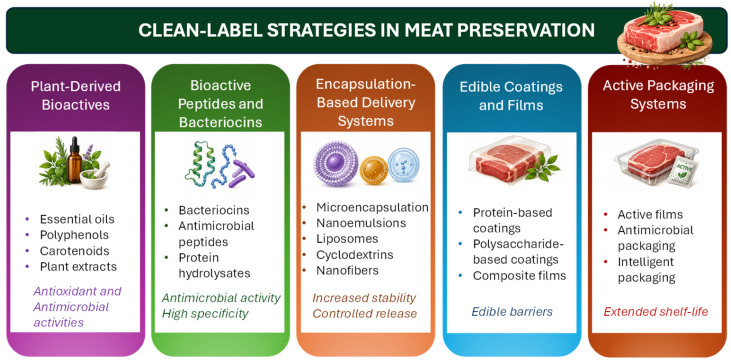
Schematic overview of clean-label preservation strategies used in meat systems.

Although the term “clean label” is not formally defined by regulatory frameworks, it has evolved as a market-driven concept reflecting consumer expectations for transparency, naturalness, and simplified ingredient lists. This trend has been partly influenced by stricter labeling requirements and increasing demand for clear product information. Clear ingredient labeling and additive classification have enhanced consumer awareness of product composition, thereby influencing purchasing decisions and accelerating the demand for simpler and more natural formulations [21]. In this context, clean-label preservation strategies in meat systems can be broadly organized around natural bioactive compounds, bioactive peptides and bacteriocins, and their integration into advanced delivery and application systems, including encapsulation-based systems, edible coatings and films, and active packaging systems (Figure 2).

Among these strategies, natural bioactive compounds including plant extracts, essential oils, phenolic compounds, and peptides, constitute one of the principal clean-label approaches for meat preservation [21]. Their properties, mechanisms of action, and technological considerations are discussed in detail in Section 5. Bioactive peptides and bacteriocins represent another important group of clean-label preservation agents and are discussed in detail in Section 5.3.

Encapsulated natural bioactives have attracted increasing attention in low-nitrite and nitrite-free meat systems, where they may help compensate for reduced antimicrobial and antioxidant protection while minimizing quality deterioration. In these products, controlled release systems may also support more stable preservation effects under reduced nitrite conditions [22]. Furthermore, certain encapsulation strategies may contribute to maintaining both color stability and microbiological quality simultaneously by protecting sensitive bioactives and enabling sustained release during storage.

Overall, the effectiveness of clean-label preservation strategies depends not only on the selection of bioactive compounds, but also on how they are stabilized, delivered, and distributed within the food matrix. Accordingly, encapsulation technologies have emerged as a key enabling approach to enhance the stability, release behavior, and functional performance of natural bioactives in meat systems. However, the successful implementation of encapsulation-based preservation strategies requires careful selection of carrier materials that provide effective bioactive protection while remaining consistent with clean-label expectations. Accordingly, food-grade biopolymers, proteins, polysaccharides, and other naturally derived materials are generally preferred for developing clean-label meat preservation systems.

A comprehensive understanding of the properties, mechanisms of action, and limitations of natural bioactives provides an essential foundation for the rational design of effective encapsulation-based preservation strategies. Accordingly, the following section provides a detailed overview of natural bioactive compounds used in meat preservation.

## 5. Natural Bioactive Compounds for Meat Preservation

Natural bioactive compounds represent one of the most extensively studied clean-label preservation strategies in meat systems due to their multifunctional roles in inhibiting microbial growth and oxidative deterioration. These compounds target key spoilage pathways through mechanisms such as membrane disruption, radical scavenging, and metal chelation. They can be broadly categorized into three major groups: plant-derived compounds (including essential oils and phenolic extracts), bioactive peptides, and bacteriocins, each exhibiting distinct mechanisms of action, technological advantages, functional properties, and application-related limitations, as discussed in the following subsections.

### 5.1. Essential Oils

Essential oils, including thymol, carvacrol, and eugenol, are among the most extensively studied natural preservatives owing to their strong antimicrobial and antioxidant properties. Their antimicrobial activity is primarily attributed to the disruption of microbial cell membranes, leading to increased permeability, leakage of intracellular components, and ultimately cell death [23]. In addition to their antimicrobial action, essential oils can contribute to oxidative stability by scavenging free radicals and interrupting lipid oxidation chain reactions, thereby delaying rancidity development in meat systems [24].

The antimicrobial effectiveness of essential oils has been widely demonstrated in various meat products. Thymol and carvacrol, for instance, have been reported to significantly reduce microbial load in fresh meat systems, while eugenol has shown effectiveness against both Gram-positive and Gram-negative bacteria [25]. Comparative studies have also shown that essential oil efficacy may differ depending on the major active constituent, applied concentration, target microorganism, and meat matrix. For example, thymol- and carvacrol-rich systems have demonstrated strong antimicrobial effects in beef, poultry, and fish products, including reductions in *Salmonella* and *E. coli* populations as well as delayed spoilage development during refrigerated storage [26,27,28]. The effectiveness of essential oils is also strongly matrix- and dose-dependent, and direct comparisons have shown that antimicrobial and antioxidant performance may vary considerably according to the food system and application level [29,30]. Synergistic combinations with biopolymers or other preservation approaches have also been demonstrated to enhance stability and prolong antimicrobial activity [31].

Despite these advantages, the application of essential oils in meat systems is significantly limited by several intrinsic and technological barriers. One of the primary limitations is their high volatility and sensitivity to environmental factors such as heat, light, and oxygen, leading to rapid loss of activity during processing and storage [4]. Moreover, their hydrophobic nature limits their dispersion in aqueous meat matrices, resulting in poor distribution and reduced antimicrobial effectiveness [32]. Interactions with meat components further complicate their functionality. Essential oils may bind to proteins and lipids within the matrix, reducing their availability for antimicrobial action [33]. In particular, distribution into lipid phases and binding interactions can significantly reduce their accessibility to microbial targets, thereby reducing their effectiveness in meat systems compared with in vitro conditions.

Another critical limitation is their strong sensory impact. Essential oils exhibit intense aroma that can easily exceed consumer acceptance thresholds, even at relatively low concentrations [34]. For example, thyme essential oil concentrations above approximately 0.02–0.05% have been associated with reduced sensory acceptance in minced turkey due to intense or solvent-like notes [30], while oregano essential oil at around 0.05% reduced odor, flavor, and overall acceptability in beef patties despite preservation benefits [35]. More broadly, several studies have shown that increasing essential oil concentrations may compromise sensory acceptability, even when oxidative stability and antimicrobial performance are improved. For example, garlic essential oil concentrations above 0.005% markedly reduced consumer acceptance of dry-cured sausage, whereas oregano essential oil at 0.05% resulted in the lowest flavor and overall acceptability scores in beef patties despite its preservative efficacy [35,36]. Therefore, essential oil concentrations should be optimized according to the meat formulation, essential oil type, and target preservation effect, as achieving effective antimicrobial activity often requires concentrations that may compromise sensory acceptability. These limitations have stimulated growing interest in encapsulation-based delivery systems as a strategy to improve essential oil stability, enhance dispersion, and maximize preservation efficacy while minimizing undesirable sensory effects.

From a regulatory and safety perspective, many essential oil components are classified as Generally Recognized as Safe (GRAS) by the U.S. Food and Drug Administration (FDA). However, regulatory evaluation frameworks differ across jurisdictions. In the European Union, natural flavoring substances are regulated under Regulation (EC) No 1334/2008 and are subject to safety assessment procedures established by the European Food Safety Authority (EFSA). These regulatory considerations should be taken into account when developing essential oil-based preservation systems intended for commercial meat applications [8].

In summary, essential oils offer significant potential as natural preservatives due to their dual antimicrobial and antioxidant functions. Compared with many non-volatile plant extracts, essential oils often exhibit stronger immediate antimicrobial activity because compounds such as thymol, carvacrol, and eugenol can rapidly disrupt microbial cell membranes. Nevertheless, their successful application in meat systems remains dependent on overcoming challenges related to stability, matrix interactions, and sensory acceptability, highlighting the importance of effective delivery strategies for clean-label preservation [8,37].

### 5.2. Polyphenols and Carotenoids

Polyphenols and carotenoids are among the most pronounced natural antioxidants explored for clean-label meat preservation, primarily due to their ability to inhibit lipid oxidation and maintain product quality during storage. Oxidative deterioration is a key factor limiting meat shelf life; therefore, these compounds play a critical role in preserving lipid stability, sensory attributes, and overall product acceptability. However, their effectiveness is strongly dependent on chemical stability, resistance to processing and storage conditions, and interactions with the meat matrix, which may limit their functionality in real systems.

#### 5.2.1. Polyphenols

Polyphenolic compounds, including flavonoids, phenolic acids, and tannins, exert their antioxidant activity through multiple complementary mechanisms. One of the primary pathways involves direct radical scavenging, where polyphenols neutralize reactive oxygen species and terminate lipid oxidation chain reactions [38]. In addition, their ability to chelate pro-oxidant metal ions, particularly Fe^2+^/Fe^3+^ is highly relevant in meat systems, where heme iron can catalyze oxidative reactions [39]. By binding these metal ions, polyphenols reduce free radical formation and slow oxidative processes. They may also inhibit oxidative enzymes such as lipoxygenase and stabilize radical species through hydrogen atom or electron donation [40]. Collectively, these mechanisms contribute to improved oxidative stability and reduced lipid deterioration in meat systems [41].

The practical effects of polyphenols in meat systems are well documented. Their incorporation has been associated with reduced lipid oxidation (e.g., thiobarbituric acid reactive substances (TBARS)), improved protein stability, and enhanced color retention, supporting extended shelf life [19,42]. For example, polyphenol-rich extracts have been reported to reduce microbial counts by up to 0.64 log CFU/g in refrigerated game meat [43], while nanoencapsulated sour tea extract at 1000 ppm decreased TBARS values by approximately 55–60% compared with the control after 9 days of refrigerated storage in chicken nuggets [44]. Additional studies have consistently shown that encapsulated polyphenols can maintain antioxidant activity for longer periods during storage, thereby improving protein stability and reducing oxidative deterioration in meat matrices [45,46]. Collectively, these findings demonstrate the potential of polyphenols, particularly in encapsulated forms, to enhance oxidative stability and preserve quality attributes during meat storage.

Despite these advantages, polyphenols are not without limitations. Their stability can be reduced under processing and storage conditions such as heat, oxygen exposure, and changes in pH [47]. Moreover, interactions with proteins and lipids may reduce their local availability, accessibility, and antioxidant efficiency, thereby limiting their effectiveness in complex meat matrices. Structural transformations under physiological, processing, or storage conditions may further influence their functional activity [48]. Compared with essential oils, phenolic compounds generally exhibit lower volatility, relatively higher stability, and less intense sensory effects; however, their antimicrobial efficacy is often more limited and strongly dependent on their chemical structure, concentration, and matrix interactions.

#### 5.2.2. Carotenoids

Carotenoids represent another critical class of natural bioactives, contributing mainly to oxidative stability and, in some cases, color preservation in meat products. Compounds such as β-carotene, lutein, and lycopene are highly effective in quenching singlet oxygen and scavenging free radicals, thereby reducing oxidative damage [49,50]. Their contribution to visual quality is primarily indirect, as the antioxidant activity of carotenoids may help delay oxidative processes associated with color deterioration during storage. However, carotenoids do not directly participate in the myoglobin redox system responsible for the characteristic color of fresh red meat.

Carotenoids also contribute to reducing oxidation-derived off-flavors by stabilizing lipid systems during storage [49]. However, similar to polyphenols, their effectiveness is influenced by their chemical structure and environmental sensitivity. Carotenoids are highly susceptible to degradation under heat, light, and oxygen exposure, resulting in loss of both color intensity and antioxidant capacity [51].

These characteristics make carotenoids particularly suitable candidates for encapsulation-based delivery systems. Their highly lipophilic nature may restrict homogeneous distribution within meat matrices, while their susceptibility to environmental degradation can substantially reduce functional effectiveness during processing and storage. Encapsulation strategies such as nanoemulsions, liposomes, and lipid-based carriers have therefore been investigated to improve carotenoid stability, enhance dispersion, and protect against environmental degradation. However, formulation design remains critical, as excessive carotenoid concentrations may introduce undesirable color deviations and negatively affect product acceptability [52]. Unlike essential oils and many phenolics, carotenoids primarily contribute to oxidative stability and color-related quality preservation rather than antimicrobial activity, making them particularly valuable in products where lipid oxidation is a major deterioration pathway.

### 5.3. Bioactive Peptides and Bacteriocins

Bioactive peptides and bacteriocins have emerged as highly effective natural antimicrobial agents for meat preservation, offering targeted activity against spoilage and selected pathogenic microorganisms. Bacteriocins represent a specific subgroup of bioactive peptides whose primary function is antimicrobial activity. In contrast, other bioactive peptides derived from protein hydrolysates may exhibit multiple biological functions, including antioxidants, antihypertensive, and, in some cases, antimicrobial effects; however, their antimicrobial activity is generally less pronounced and less specific than that of bacteriocins. Compared with many plant-derived bioactives, bacteriocins exhibit more specific modes of action and are generally more effective against Gram-positive bacteria, mainly through disruption of membrane integrity and, in some cases, interference with cell wall biosynthesis [53]. Consequently, they provide potent activity against specific microbial groups, although their broad-spectrum applicability remains limited. Nisin is the most extensively studied and commercially applied bacteriocin, owing to its strong antimicrobial efficacy, well-established safety profile, and broad regulatory acceptance [54].

In addition to nisin, several other bacteriocins have demonstrated potential for meat preservation applications. Pediocin PA-1/AcH is particularly effective against *Listeria monocytogenes* and has been incorporated into meat products and packaging systems to improve microbiological safety [55]. Enterocin AS-48 exhibits broad antimicrobial activity against spoilage and pathogenic Gram-positive bacteria and has shown potential in refrigerated meat preservation [56]. Lacticin 3147 has also attracted attention due to its dual-peptide mode of action and effectiveness against foodborne pathogens, including *Listeria* species [57]. Although these bacteriocins have generally received less commercial attention than nisin, they highlight the diversity of bacteriocin-based preservation strategies available for meat systems.

The antimicrobial mechanisms of bacteriocins vary depending on their class and molecular structure. For nisin and some other lantibiotics, binding to lipid II, a key precursor in bacterial cell wall biosynthesis, represents an important mechanism. This interaction induces pore formation in the cytoplasmic membrane, leading to leakage of intracellular components and rapid cell death [58]. Other bacteriocins, including several class II peptides, primarily act by permeabilizing the bacterial membrane and disrupting membrane potential [59]. Gram-negative microorganisms generally exhibit higher resistance due to the presence of an outer membrane barrier [60].

Nevertheless, bacteriocins have demonstrated considerable potential in meat preservation applications through direct incorporation, surface treatments, and integration into edible films or coatings [61]. Such applications have shown significant reductions in microbial load and extended shelf life in meat products. Combination strategies (e.g., Nisin with EDTA) further enhance activity by increasing membrane permeability in Gram-negative bacteria, thereby broadening antimicrobial effectiveness [62].

Despite these advantages, several limitations restrict their application in complex meat systems. Interactions with meat components, including proteins and lipids, can reduce their availability and antimicrobial activity by limiting diffusion and accessibility to target microorganisms [63]. Additionally, their relatively narrow antimicrobial spectrum, primarily against Gram-positive bacteria, restricts their broad applicability. Environmental factors such as pH, ionic strength, and enzymatic degradation may further affect their stability and performance [60]. Diffusion limitations in heterogeneous and high-fat matrices can also lead to uneven distribution and localized antimicrobial activity [58].

Compared to plant-derived bioactives, peptide-based systems provide higher specificity and potent antimicrobial activity against susceptible microorganisms, but their performance may be limited by diffusion constraints, interactions with food components, enzymatic degradation, and relatively high production costs. Conversely, plant-derived bioactives exhibit broader multifunctionality, although their antimicrobial effects are generally less specific and less potent. These constraints make encapsulation an attractive strategy to improve stability, distribution, and overall antimicrobial effectiveness in meat systems.

### 5.4. Plant Extracts and Emerging Bioactives

Plant extracts represent a broader category of natural bioactives than essential oils or purified phenolic compounds. In the context of this review, this section focuses on multicomponent botanical extracts and agro-industrial by-product extracts that contain complex mixtures of phenolics, terpenoids, organic acids, pigments, and other bioactive constituents. Unlike isolated essential oil fractions or purified phenolic compounds discussed in previous sections, these extracts derive their functionality from the combined and potentially synergistic activity of multiple components, which may contribute simultaneously to antioxidant, antimicrobial, and quality-preserving effects in meat systems.

The antioxidant activity of plant extracts is largely attributed to phenolic compounds, which act through radical scavenging and metal chelation mechanisms, thereby enhancing oxidative stability [64]. In parallel, plant-derived compounds contribute to microbial inhibition through membrane disruption, enzyme interference, and metabolic inhibition, providing a broad-spectrum preservation effect [65].

Plant extracts have been applied in various forms within meat systems, including direct incorporation and use in coatings or films for targeted surface protection [14]. Extracts from herbs such as oregano, rosemary, and green tea have shown preservation potential by improving oxidative stability, microbial quality, shelf life, and sensory attributes [66]. In addition, growing interest has been directed toward bioactives derived from agricultural and food-processing by-products, such as fruit peels, seeds, and vegetable residues, which represent sustainable and cost-effective alternatives rich in bioactive compounds, aligning with circular economy principles [67,68].

The effectiveness of natural bioactives in meat systems shows considerable variability across studies. While polyphenols and carotenoids are generally associated with improved oxidative stability, and bacteriocins often provide stronger antimicrobial activity against susceptible microorganisms, preservation outcomes are strongly influenced by bioactive composition, concentration, meat matrix characteristics, storage conditions, and application strategy. Moreover, many natural bioactives share common limitations, including instability, variability in composition, limited availability within the food matrix, and potential sensory impacts, which may further influence their performance in meat systems [3]. Interactions with proteins and lipids, diffusion limitations, and differences in release behavior may substantially affect bioactive availability and functionality in real food systems.

Consequently, preservation outcomes depend not only on the intrinsic properties of the bioactive compounds but also on the characteristics of the meat matrix and the selected delivery strategy. Therefore, results obtained under model conditions are not always directly transferable to commercial meat products, highlighting the importance of tailoring preservation strategies to specific product characteristics and processing conditions. In this context, encapsulation does not invariably result in superior preservation performance. For example, in refrigerated minced meat, free *Thymus schimperi* essential oil exhibited greater in situ antibacterial activity than both spray-dried and freeze-dried encapsulated formulations, despite the improved stability provided by encapsulation [69]. Similarly, Boualem et al. [70] developed a liposomal nisin delivery system designed to release nisin only after liposome melting above 37 °C. While this approach protected nisin from inactivation in raw meat and enabled antimicrobial activity during cooking, it also illustrates that controlled-release systems may not be suitable when immediate antimicrobial activity is required during refrigerated storage. Collectively, these studies demonstrate that encapsulation performance depends not only on carrier design but also on matching release kinetics to the preservation objective, meat matrix characteristics, and processing conditions.

## 6. Encapsulation Technologies for Bioactive Delivery Systems

Encapsulation technologies offer diverse approaches to improve the stability, release behavior, and functionality of natural bioactives. The selection of an appropriate system depends on the physicochemical properties of the bioactive compound, the characteristics of the meat matrix, and the desired release profile. In this context, encapsulation plays a critical role not only in protecting bioactives but also in governing their interaction with complex food systems.

As summarized in Table 1, the major limitations associated with the direct use of natural bioactives in meat systems can be directly addressed through specific encapsulation functionalities.

In addition to the intrinsic properties of encapsulation systems, their performance in meat products is strongly influenced by interactions with the surrounding food matrix. For example, hydrophobic bioactives may preferentially partition into lipid-rich regions of the matrix, reducing their accessibility to microbial targets, whereas interactions with proteins can hinder diffusion and alter release behavior. As a result, delivery systems that perform effectively in simplified model environments may exhibit different release kinetics and preservation efficacy in real meat products. Consequently, preservation performance is not determined solely by encapsulation efficiency or carrier design, but also by the extent to which bioactive compounds remain available and functionally active within the meat matrix. Understanding these matrix-dependent interactions is therefore essential for the rational design of effective delivery systems for meat preservation.

This section critically examines encapsulation strategies with emphasis on structure–function relationships, release behavior, and applicability in meat systems.

### 6.1. Microencapsulation Approaches: From Conventional to Scalable Systems

Microencapsulation represents one of the most widely applied strategies for improving the stability and functionality of natural bioactives in meat systems, particularly due to its scalability and industrial compatibility. Among available techniques, spray drying, freeze-drying, and coacervation are among the most commonly used approaches, each offering distinct advantages and limitations in terms of encapsulation efficiency, bioactive protection, release behavior, cost-effectiveness, and scalability.

Spray drying is widely used due to its operational simplicity, scalability, and low cost. It involves atomization of a bioactive-containing liquid into hot air, resulting in rapid solvent evaporation and formation of dry microcapsules [79]. Typical wall materials include carbohydrates (maltodextrin, gum arabic), proteins, and modified starches, which enhance structural stability and protect bioactives [80]. Encapsulation efficiency typically ranges between 60–90%, depending on formulation parameters, wall material composition, inlet/outlet temperature, feed properties, and core-to-wall ratio [81]. However, spray drying presents limitations, particularly for volatile and heat-sensitive compounds, where high temperatures may lead to degradation and loss of activity [82]. Spray-dried particles may also exhibit rapid release due to their porous structure, limiting sustained activity [9].

Freeze-drying (lyophilization) is another widely used microencapsulation approach, particularly for heat-sensitive bioactives such as bacteriocins, polyphenols, and certain plant-derived compounds. Unlike spray drying, freeze-drying removes water through sublimation under low-temperature and low-pressure conditions, thereby minimizing thermal degradation and preserving bioactive functionality [83]. As a result, this method can provide high bioactive retention and encapsulation efficiency, especially in formulations where thermal protection is critical. However, freeze-drying is associated with high processing costs, long drying times, and relatively low production throughput. In addition, the resulting particles often exhibit irregular morphology and broader size distributions than spray-dried microcapsules, which may affect powder handling and large-scale processing. These limitations restrict its industrial applicability despite its superior ability to preserve sensitive bioactives [81].

Coacervation is a phase separation-based technique involving electrostatic interactions between oppositely charged biopolymers, forming dense microcapsules. Compared to spray drying, it may provide higher encapsulation efficiency, often exceeding 90% under optimized conditions, and better protection of sensitive compounds [84]. The dense structure can enable controlled and sustained release, which is particularly beneficial for prolonged activity in meat systems [79]. However, the method is more complex, sensitive to environmental conditions (pH, ionic strength), and less scalable, limiting industrial application [85].

Encapsulation performance is strongly influenced by formulation parameters such as wall material, particle size, core-to-wall ratio, and structural organization [86]. Smaller particles may improve dispersion and can also promote faster release due to their higher surface area [87]. In meat systems, interactions with proteins, lipids, and water significantly influence release behavior and bioactive availability [10,88,89]. Therefore, encapsulation efficiency alone does not adequately reflect functional performance; rather, controlled release behavior and matrix interactions are the primary determinants of efficacy.

From a practical perspective, spray drying offers clear advantages in terms of scalability, cost-effectiveness, and industrial feasibility, making it suitable for routine incorporation of relatively stable plant extracts or bioactives into processed meat products. Freeze-drying is more appropriate for heat-sensitive compounds and high-value formulations where bioactive retention is prioritized, although its cost and processing limitations restrict broad industrial use. Coacervation, in contrast, may be more suitable when maximum protection of sensitive, volatile, or oxidation-prone compounds and prolonged release are required. This comparison highlights that the selection of encapsulation systems should be guided by application-specific requirements, particularly when balancing industrial feasibility with functional performance in real meat matrices.

### 6.2. Nano-Delivery Systems: Enhancing Bioavailability and Antimicrobial Efficiency

Nano-delivery systems have emerged as advanced encapsulation platforms that address key limitations of conventional microencapsulation, particularly in terms of local availability, dispersion, and antimicrobial efficiency. Their reduced particle size provides a high surface area-to-volume ratio, enhancing interaction with microbial cells and improving distribution within complex meat matrices. Among these systems, nanoemulsions, nanoliposomes and solid lipid nanoparticles (SLNs) are the most widely investigated systems for meat preservation applications.

Nanoemulsions, typically oil-in-water (O/W) systems stabilized by surfactants or biopolymers, offer high kinetic stability and improved solubilization of hydrophobic compounds such as essential oils. Their small droplet size enables uniform dispersion, enhances antimicrobial activity at lower concentrations, and increases the accessibility of hydrophobic bioactives to microbial membranes and lipid phases susceptible to oxidation [79,80].

Nanoliposomes are vesicular systems composed of phospholipid bilayers capable of encapsulating both hydrophilic and hydrophobic compounds. This dual functionality enables the design of multifunctional delivery systems. In meat systems, nanoliposomes protect sensitive bioactives from degradation and enable controlled release over time, thereby enhancing preservation performance [90]. Their structural similarity to biological membranes facilitates interaction with microbial cells and improves the delivery of encapsulated bioactives, thereby enhancing antimicrobial efficacy [91].

SLNs represent an important lipid-based delivery system composed of biocompatible solid lipid matrices capable of entrapping lipophilic bioactive compounds. The solid lipid core provides protection against oxidation, light exposure, and chemical degradation while enabling controlled release during storage [92]. Compared with conventional emulsions, SLNs generally exhibit improved physical stability and are particularly suitable for the delivery of hydrophobic antioxidants and essential oil components. Their ability to maintain bioactive stability under low-temperature conditions has attracted interest in frozen meat applications. For example, SLN-based systems have been reported to improve oxidative stability and extend shelf life in frozen beef products during long-term storage [4,93].

Rather than directly disrupting microbial membranes, nanoscale delivery systems enhance the dispersion, contact frequency, and effective local concentration of encapsulated bioactives at microbial surfaces, particularly for hydrophobic compounds. Antimicrobial action itself is primarily governed by the released bioactive compounds, such as essential oil components or bacteriocins, which may disrupt membrane integrity, form pores, or interfere with microbial metabolic processes [89]. Encapsulation efficiency in nano-delivery systems is generally high, often exceeding conventional systems due to improved entrapment [86]. These systems also enable better control over release kinetics, supporting sustained delivery during storage.

Despite these advantages, nano-delivery systems present several challenges. Physical instability (e.g., aggregation, coalescence, phase separation) may occur under varying temperature, pH, and ionic conditions [79]. Similarly, nanoliposomes may exhibit leakage or structural instability, affecting bioactive retention and release. In addition, interactions with the meat matrix, as discussed earlier, can influence nanoparticle behavior, potentially leading to aggregation or altered release profiles [88]. For example, lipid-rich environments may favor the retention of hydrophobic bioactives, while protein interactions may hinder diffusion and reduce antimicrobial activity. Therefore, nano-carriers that demonstrate excellent dispersion, encapsulation efficiency, and antimicrobial performance in model systems may behave differently in real meat products, where proteins, lipids, salts, and water phases can alter nanoparticle distribution, bioactive partitioning, and release kinetics.

In addition to technical challenges, regulatory and safety considerations remain critical for the application of nano-delivery systems in food products. Consumer perception and regulatory requirements may influence their adoption in commercial meat products. These considerations are particularly important in clean-label-oriented preservation strategies, where the use of nanoscale delivery systems must be balanced with expectations for naturalness, transparency, and consumer acceptance.

Overall, nano-delivery systems enhance dispersion, local availability, antimicrobial efficiency, and controlled release of bioactives, offering important advantages over conventional systems. Nanoemulsions are particularly suitable for improving the delivery of volatile essential oils and other hydrophobic antimicrobials, making them advantageous for surface microbial control. In contrast, nanoliposomes offer greater flexibility for multifunctional preservation strategies because they can simultaneously carry hydrophilic and lipophilic bioactives. SLNs are especially promising for lipophilic antioxidants and essential oil components requiring improved protection against oxidation and controlled release. Therefore, the selection of a nano-delivery platform should be guided by the bioactive compound, target spoilage pathway, product matrix, storage conditions, and regulatory or consumer acceptance considerations.

### 6.3. Molecular Encapsulation and Complexation Systems

Molecular encapsulation and complexation systems represent a distinct class of delivery strategies. These systems are based on inclusion complex formation, typically involving cyclodextrins. They offer advantages in stabilizing bioactive compounds and modulating their release. Through reversible complex formation, bioactive molecules can be protected from degradation while their solubility and overall stability are improved.

Cyclodextrins, including α-, β-, and γ-cyclodextrin, are widely used due to their hydrophilic outer surface and hydrophobic internal cavities, enabling the encapsulation of hydrophobic compounds such as essential oils and phenolics. This property improves the solubility, stability, and retention of volatile or poorly water-soluble compounds in meat systems [79]. Molecular encapsulation can also reduce volatility and protect bioactives from environmental degradation caused by oxygen, light, or heat [80]. In addition, these systems may help to reduce sensory impact by controlling release and masking strong flavors, particularly in essential oils [81]. Encapsulation efficiency and complex stability depend on molecular compatibility and environmental conditions (pH, temperature, moisture). Although the loading capacity is relatively lower than in conventional systems, their high binding specificity ensures effective stabilization [86]. The reversible nature of inclusion complexes enables controlled release, which can be triggered by dilution, changes in the surrounding environment, or interactions with food components.

In meat systems, proteins, lipids, and other matrix constituents may compete with cyclodextrins for binding to bioactives, influencing release behavior and potentially leading to premature release or non-uniform distribution within the matrix [89]. Therefore, although molecular encapsulation improves the stability and retention of volatile compounds such as essential oils, its effectiveness may be limited by relatively low loading capacity and interactions within complex food matrices. Nevertheless, cyclodextrin-based delivery systems have demonstrated promising performance in meat preservation. For example, alginate-based edible coatings containing oregano essential oil/cyclodextrin inclusion complexes improved essential oil stability and enabled sustained release, resulting in reduced lipid oxidation, TVB-N, and microbial growth during refrigerated storage of chicken breast [94].

Overall, cyclodextrin inclusion complexes are particularly advantageous for highly volatile essential oil components and other poorly water-soluble compounds because they can reduce evaporation losses, improve stability, and mitigate undesirable sensory impacts. However, their relatively low loading capacity may limit their use in applications requiring high bioactive concentrations. Therefore, cyclodextrin-based systems are most suitable for applications in which stabilization, sensory masking, and controlled localized release are prioritized over high loading capacity. These limitations can be further addressed through advanced structuring and fabrication approaches, as discussed in the following section.

### 6.4. Advanced Fabrication Techniques for Structured Delivery Systems

Advanced fabrication techniques refer to modern methods used to design and structure encapsulation systems in a controlled manner. These techniques allow the formation of delivery systems with defined structures at the micro- and nanoscale [95]. Unlike conventional encapsulation systems, which mainly focus on protecting bioactives within relatively simple carrier structures, advanced fabrication approaches enable more precise control over structural organization, release behavior, and responsiveness to environmental conditions [86]. By controlling the structure, it becomes possible to regulate how bioactive compounds are distributed and released within food systems. This is particularly important in meat matrices, where interactions with proteins and lipids can strongly affect performance. As a result, these approaches may improve the stability, functionality, and effectiveness of bioactive compounds compared to conventional encapsulation methods [96]. Accordingly, this section introduces electrospinning, electrospraying, multilayer assembly, and stimuli-responsive systems and discusses their design and applications in meat preservation.

#### 6.4.1. Electrospinning

Electrospinning is a widely used technique for developing nanofibrous delivery systems with high surface area, tunable porosity, and controlled release properties [97,98]. These characteristics support efficient encapsulation and sustained release of bioactives. The process involves applying electrostatic forces to polymer solutions to produce ultrafine fibers that entrap bioactives within or on the fiber matrix. Encapsulation performance depends on factors such as voltage, polymer concentration, and material properties [99,100]. Biopolymers such as zein, chitosan, and gelatin are commonly used to improve biocompatibility and control mechanical and degradation properties [101].

In meat preservation, electrospun nanofibers are mainly used in active packaging systems, where they serve as reservoirs for antimicrobial and antioxidant agents. Their high surface area enhances interaction with microorganisms, while the fibrous structure supports sustained release at the product surface [102,103]. In meat applications, chitosan–gelatin nanofibers containing *Ziziphora clinopodioides* essential oil and *Heracleum persicum* extract have been applied in beef sausages, resulting in reduced microbial growth and lipid oxidation, improved sensory quality, and extended shelf life during storage [104]. Electrospun chitosan/PVA nanofibers loaded with Artemisia essential oil also reduced peroxide value, total amount of volatile basic nitrogen (TVB-N), and microbial counts in minced beef [105]. In addition, these systems can be designed to respond to environmental factors such as humidity and temperature, enabling adaptive release [106]. However, challenges remain related to scalability, cost, and process stability, particularly in maintaining uniform fiber structure at industrial scale [97].

#### 6.4.2. Electrospraying

Electrospraying enables the production of micro- and nanoparticles with controlled size and morphology, offering an alternative to fibrous systems [97]. These particles allow targeted delivery and improved dispersion in food matrices. System performance depends on formulation and processing parameters, including solvent composition and polymer type, flow rate, voltage, and collector distance [107]. Particle size plays a key role in release behavior, with smaller particles generally enabling faster release and larger structures support sustained delivery. This makes electrospraying particularly suitable for delivering bioactives in meat systems, especially when uniform dispersion and controlled release are required [108]. In meat applications, nanoencapsulated garlic essential oil particles with sizes in the nanometer range improved microbial stability, sensory quality, and shelf life of refrigerated hamburgers compared with free essential oil [109]. Chitosan coatings containing nanoencapsulated *Satureja montana* essential oil also reduced total viable counts and lipid oxidation in beef [110]. However, stability challenges such as aggregation and environmental sensitivity must be considered [111].

#### 6.4.3. Layer-by-Layer and Multilayer Systems

Layer-by-layer assembly involves the sequential deposition of oppositely charged polymers to form structured multilayer systems [112]. These systems provide improved control over barrier properties and release kinetics. Multilayer structures act as diffusion barriers, enabling sustained and controlled release of bioactives [113]. They can incorporate proteins, polysaccharides, and lipids, allowing multifunctional system design.

In meat systems, layer-by-layer systems improve stability and prolong functional activity. For example, in multilayer systems, chitosan films containing rosemary oil nanoemulsions reduced *Pseudomonas* spp. growth and maintained acceptable peroxide and TVB-N values in minced beef [114]. Gelatin/chitosan/carboxymethyl cellulose (CMC) multilayer films containing selenium nanoparticles and beetroot extract also extended beef shelf life by reducing microbial growth and oxidative deterioration [115]. However, process complexity and scalability remain important limitations [116].

#### 6.4.4. Smart (Stimuli-Responsive) Systems

Stimuli-responsive systems enable controlled release or functional responses triggered by environmental factors such as pH, temperature, and moisture [117]. In principle, pH-responsive delivery systems may modulate bioactive release in response to pH changes associated with food spoilage. However, direct evidence demonstrating pH-triggered antimicrobial release with quantified release kinetics and measurable microbial inhibition in real meat matrices remains limited. Although some studies have investigated pH-responsive systems in real meat products, the available evidence remains limited, and most research has primarily focused on freshness monitoring through pH-dependent color changes. In addition, release behavior is still frequently characterized in buffer or model systems rather than being directly quantified in meat products together with measurable antimicrobial outcomes [118]. Therefore, the antimicrobial relevance of pH-triggered release in actual meat systems should be interpreted cautiously and requires further validation under realistic storage conditions. In smart packaging applications, pH-responsive multilayer films containing beetroot extract showed visible color changes during beef spoilage while also providing antimicrobial and antioxidant protection [115]. Similarly, biodegradable films containing roselle anthocyanins were successfully used to monitor pork freshness through pH-dependent color transitions associated with spoilage development [119]. However, formulation complexity, cost, regulatory constraints, and the limited availability of real-matrix release data currently restrict the industrial application of these systems [120].

Overall, the suitability of advanced fabrication technologies depends on the intended preservation objective. Electrospinning is particularly advantageous for long-term and surface-targeted release through active packaging systems. Electrospraying offers greater flexibility for direct incorporation into food matrices where homogeneous dispersion is required. Layer-by-layer systems provide superior control over release kinetics and barrier properties, whereas stimuli-responsive systems are most appropriate for intelligent packaging applications requiring condition-dependent monitoring or release, although their antimicrobial release behavior in real meat matrices still requires further validation. Despite their functional advantages, these technologies currently face greater challenges in industrial scalability, process reproducibility, regulatory acceptance, and production costs than conventional encapsulation approaches.

#### 6.4.5. Challenges

The effectiveness of advanced fabrication systems depends on the integration of structural design with functional requirements. Hybrid systems combining multiple approaches (e.g., electrospinning and multilayer systems) can enhance performance through complementary effects [121]. However, system design must consider material selection, structural parameters, release behavior, and compatibility with the meat matrix, as interactions with proteins, lipids, and water strongly influence release performance and bioactive stability, requiring application-specific optimization [122]. From an industrial perspective, scalability, cost, and regulatory compliance remain key challenges, which may limit widespread adoption of these systems.

Based on the current literature, no single encapsulation system can be considered universally superior. Instead, system selection should be driven by the characteristics of the bioactive compound, the target spoilage pathway, and the intended preservation objective. Cyclodextrin complexes appear most suitable for volatile essential oils, nanoemulsions for hydrophobic antimicrobial compounds requiring rapid interaction with microbial cells, nanoliposomes for multifunctional delivery of mixed bioactives, coacervation systems for prolonged release and protection of sensitive compounds, and electrospun systems for active packaging applications requiring sustained surface release. For industrial-scale implementation, spray drying remains the most practical and economically feasible approach, whereas advanced fabrication technologies offer greater functional control but currently face scalability challenges.

### 6.5. Structure–Function Relationships in Encapsulation Systems

The performance of encapsulation systems in meat preservation depends on structural design and functional properties. Parameters such as particle size, morphology, matrix composition, and interfacial properties collectively determine stability, release behavior, and bioactivity of encapsulated compounds. This structure–function relationship has been demonstrated in several meat applications. For instance, sodium alginate nanoparticles containing lemon verbena essential oil with smaller particle size (~150–200 nm) and higher surface charge stability improved antimicrobial activity and oxidative stability in refrigerated beef, leading to lower microbial counts and reduced lipid oxidation [123]. Similarly, garlic essential oil nanoliposomes with nanoscale particle size and good colloidal stability reduced microbial growth and improved the microbiological quality of refrigerated beef hamburgers, while composite essential oil microcapsules exhibiting encapsulation efficiencies above 80% and sustained release behavior reduced microbial growth, TBARS, and TVB-N values in chilled pork, extending shelf life by approximately six days [109,124]. Together, these findings demonstrate that encapsulation performance is not determined by particle size alone, but rather by the combined effects of particle structure, carrier composition, release behavior, and interactions with the meat matrix. System performance is further influenced by environmental conditions and the physicochemical characteristics of the meat matrix [125,126]. Microscale systems can provide enhanced physical stability and protection, whereas nanoscale systems may improve dispersion and antimicrobial efficiency due to their higher surface area. However, smaller particles often exhibit faster release due to shorter diffusion pathways and greater surface exposure [127]. Therefore, optimal design requires balancing enhanced bioactivity with controlled release [128].

Encapsulation matrix composition directly influences both protection and release behavior. Polysaccharide-based systems (e.g., alginate, chitosan) can provide structured diffusion barriers, while protein-based systems enable interactions with both hydrophilic and hydrophobic bioactives. Lipid-based carriers are particularly effective for hydrophobic compounds, improving retention and oxidative stability [129].

Encapsulation efficiency reflects loading capacity but does not always indicate functional performance. Release behavior, governed by structural features such as matrix density and porosity, is a key determinant of antimicrobial and antioxidant activity [87,120]. Interactions with the meat matrix further influence system performance. Proteins, lipids, and ionic components affect structural integrity, diffusion pathways, and bioactive availability, either enhancing retention or restricting diffusion depending on system composition [130].

The interfacial layer surrounding encapsulated bioactives plays an important role in their stability and release. Well-structured interfaces can improve the retention of bioactives, control their diffusion, and enable controlled delivery, especially in complex food matrices [117]. Stimuli-responsive systems release bioactives in response to environmental factors such as pH, temperature, and moisture, allowing release to adapt to storage conditions [131]. Encapsulation systems should therefore be designed according to application needs to achieve both rapid release for antimicrobial action and sustained release for long-term preservation. Overall, the selection of encapsulation systems in meat applications should be guided by the alignment between system design and functional requirements, considering the type of bioactive compound, desired release profile, processing conditions, and economic feasibility.

Across the reviewed studies, no single encapsulation system consistently outperformed all others under every application condition. Reported differences in antimicrobial and antioxidant performance are frequently associated with variations in bioactive properties, carrier composition, release kinetics, meat matrix characteristics, and storage conditions. While nanoscale systems often demonstrate superior dispersion and bioactive accessibility, these advantages do not always translate into improved preservation efficacy in real meat products, where matrix interactions may alter release behavior and bioactive availability. Similarly, systems providing stronger protection may exhibit slower release rates that are not always aligned with spoilage dynamics. These observations highlight that encapsulation performance is governed by the balance between protection, release behavior, and matrix compatibility rather than by encapsulation technology alone.

## 7. Applications and Functional Performance in Meat Systems

Encapsulated bioactives can be applied in meat systems through multiple strategies, including direct incorporation into formulations, surface treatments, and integration into active packaging systems. These approaches differ not only in terms of distribution and release control but also in their interaction with the meat matrix, which ultimately governs preservation efficiency. Direct incorporation can promote relatively uniform distribution of bioactives throughout the product, enabling internal protection against lipid oxidation and microbial growth [9]. However, this approach may also result in rapid release and interactions with proteins and lipids, reducing local bioactive availability and functional activity [10].

Surface-based strategies, such as edible coatings and films, provide more targeted protection, particularly against surface contamination where spoilage is most likely to occur. These systems act as diffusion barriers while enabling gradual release of encapsulated compounds, thereby improving antimicrobial efficiency and oxidative stability [72]. However, their effectiveness may be influenced by coating uniformity, adhesion properties, and mechanical stability during storage.

Active packaging systems represent another important application strategy, in which encapsulated bioactives are incorporated into packaging materials to allow controlled release during storage. Examples include antimicrobial films containing essential oils, nanofiber-based packaging systems, and cyclodextrin-functionalized materials, which can enhance shelf life while minimizing direct interaction with the food matrix [6,132]. This reduced interaction supports more consistent release behavior compared to direct incorporation, making packaging-based systems particularly suitable for long-term and surface-targeted preservation. To provide a structured overview of recent research and highlight application-specific outcomes in real meat systems, representative studies employing different encapsulation strategies are summarized in Table 2. Although most of the included studies were conducted in real meat products or meat-based systems, the level of validation varies from laboratory-scale product studies to pilot-scale investigations, while evidence from industrial-scale applications remains limited.

A closer examination of the studies summarized in Table 2 reveals several important trends regarding the practical performance of encapsulated bioactives in meat preservation. Essential oil-based nanoemulsions constitute one of the most frequently investigated delivery systems and consistently demonstrate improvements in microbial stability, oxidative resistance, and shelf-life extension across different meat products. Notably, several nanoemulsion-based formulations achieved reductions of approximately 2–3 log CFU/g in microbial populations and substantially decreased lipid oxidation during refrigerated storage. Chitosan-based systems, including coatings and nanoparticle carriers, provided dual functionality by combining the intrinsic antimicrobial properties of chitosan with the controlled release of encapsulated bioactives, resulting in enhanced oxidative stability and color preservation. In contrast, lipid-based carriers such as solid lipid nanoparticles appeared particularly advantageous for long-term frozen storage applications, where they improved antioxidant retention and prolonged shelf life more effectively than free bioactive compounds. Furthermore, emerging approaches such as electrospun delivery systems and intelligent packaging materials not only delayed spoilage but also contributed additional functionalities, including freshness monitoring and sustained release behavior.

Overall, the studies presented in Table 2 suggest that preservation efficacy is influenced not only by the nature of the bioactive compound but also by the compatibility between the encapsulation system, storage conditions, and the targeted spoilage mechanism. However, not all studies reported equivalent levels of improvement. In some cases, the benefits of encapsulation were modest or strongly dependent on storage conditions, bioactive concentration, and meat matrix characteristics. For example, preservation outcomes reported in minced meat products were not always comparable to those observed in more structured meat systems, suggesting that matrix characteristics can substantially influence encapsulation performance. These observations indicate that system efficacy remains highly application-dependent and is influenced by matrix-specific factors such as fat content, pH, moisture level, and product structure.

The studies summarized in Table 2 should therefore not be interpreted as directly interchangeable across meat products. Lipid-rich matrices may retain hydrophobic bioactives such as essential oil components, whereas pH variations can alter carrier swelling, release kinetics, and local bioactive availability. Consequently, encapsulation performance should be evaluated in relation to the specific characteristics of each meat matrix rather than solely on the basis of encapsulation technology.

As shown in Table 2, different encapsulation systems provide distinct functional advantages depending on the preservation objective and storage conditions. Nanoemulsion-based systems are particularly effective for improving the dispersion and local availability of hydrophobic bioactives, whereas chitosan-based coatings and films provide additional antimicrobial protection through barrier formation and controlled release. Lipid-based carriers such as solid lipid nanoparticles appear more advantageous under long-term frozen storage conditions due to their ability to protect oxidation-sensitive compounds. These observations highlight that different delivery systems may be advantageous under different preservation scenarios depending on carrier structure, release behavior, and storage conditions. The effectiveness of these application strategies is closely linked to release mechanisms, which are governed by diffusion, matrix degradation, and environmental triggers such as pH, temperature, and moisture. In meat systems, release behavior is further influenced by the complex composition of the matrix, including proteins, lipids, and ionic components, which can alter diffusion pathways and bioactive stability [144]. Consequently, predicting release behavior in real meat systems remains challenging and requires system-specific evaluation. Importantly, preservation performance is strongly influenced by the extent to which release kinetics correspond to the progression of spoilage processes under actual storage conditions.

A critical limitation in current research is the gap between in vitro release studies and actual performance in real food systems. This discrepancy appears particularly relevant for systems whose release behavior is strongly influenced by matrix interactions. While nanoemulsions and other dispersed systems often demonstrate rapid release and antimicrobial activity under simplified laboratory conditions, diffusion restrictions, protein binding, and lipid partitioning within meat matrices may reduce the effective concentration of released bioactives at the target site. Consequently, release profiles obtained in buffer systems may overestimate preservation performance under real storage conditions. Simplified models often fail to capture the complexity of meat matrices, leading to overestimation of bioactive efficacy. This gap highlights the need to design studies that better reflect real processing and storage conditions and to validate release behavior together with antimicrobial, oxidative, and sensory outcomes in real meat products [145].

From a functional perspective, encapsulated bioactives have been shown to significantly improve oxidative stability and microbial safety in meat products. However, their impact on sensory attributes remains a key challenge. Changes in flavor, aroma, and texture must be carefully controlled, as excessive release or high concentrations of bioactives may negatively affect consumer acceptance [146]. Therefore, optimizing release profiles and dosage levels is essential to balance preservation efficacy with sensory quality.

A major challenge in the practical application of natural bioactives is achieving an appropriate balance between preservation efficacy and sensory acceptability. Essential oils and plant-derived extracts often exhibit strong aromas, flavors, or color characteristics that may negatively influence consumer perception when applied at concentrations required for effective antimicrobial or antioxidant activity. In this context, encapsulation serves not only as a protective and controlled-release strategy but also as a sensory masking tool. By reducing the immediate exposure of volatile compounds and regulating their release, encapsulation systems can mitigate undesirable sensory effects while maintaining functional performance. Several studies summarized in Table 2 reported improved sensory quality or consumer acceptability when bioactives were applied in encapsulated form compared with their free counterparts. However, sensory evaluation was not consistently included across all studies, making it difficult to establish general conclusions regarding consumer acceptance. Furthermore, the optimal concentration required to achieve microbial and oxidative stability does not always coincide with the concentration preferred by consumers. Therefore, future research should place greater emphasis on dose–efficacy–acceptability relationships and incorporate standardized sensory evaluation protocols alongside technological performance assessments to facilitate industrial adoption.

Taken together, the reviewed studies indicate that encapsulation performance is governed not by the encapsulation technology alone but by the interaction between carrier structure, release behavior, bioactive properties, and application strategy. For example, cyclodextrin inclusion complexes are particularly advantageous for stabilizing volatile compounds, nanoemulsions improve the dispersion and antimicrobial effectiveness of hydrophobic bioactives, coacervation systems provide enhanced protection and prolonged release, and electrospun structures are especially suitable for sustained surface delivery through active packaging. These observations suggest that preservation efficacy is governed by the alignment between carrier structure, release kinetics, and the dominant spoilage mechanism rather than by the encapsulation technology itself. Consequently, the optimal encapsulation strategy should be selected according to the preservation objective and the physicochemical characteristics of both the bioactive compound and the target meat system, highlighting that system-level compatibility is a stronger determinant of performance than the choice of a specific delivery technology alone.

In conclusion, the success of encapsulation strategies in meat systems depends on the alignment between application method, release behavior, bioactive properties, and product characteristics, highlighting the need for careful system selection based on specific processing and storage conditions.

## 8. Industrial Translation and Future Perspectives

Encapsulated natural bioactives offer significant opportunities for improving meat preservation; however, their successful translation from laboratory-scale research to industrial applications remains challenging. Despite promising results under controlled conditions, large-scale implementation is often limited by technological, economic, and regulatory constraints [147].

From a technological perspective, encapsulation technologies differ substantially in their level of industrial maturity. Spray drying remains one of the most established and commercially feasible approaches due to its high throughput, cost-effectiveness, and compatibility with existing food-processing infrastructure. Nanoemulsion-based systems also show considerable applied potential, particularly for improving the dispersion and delivery of hydrophobic bioactives; however, their industrial implementation still requires further optimization in terms of formulation stability, regulatory acceptance, and consumer perception. In contrast, emerging structured delivery technologies, such as electrospinning, layer-by-layer assembly, and stimuli-responsive systems, remain largely at laboratory or pilot scale for food applications, with unresolved challenges related to production throughput, solvent safety, process stability, reproducibility, and scale-up. Therefore, industrial translation should be evaluated according to technology readiness, distinguishing mature scalable methods from emerging systems that provide high functional precision but currently have lower commercial readiness [4,90].

The integration of encapsulated bioactives into meat systems also requires compatibility with existing processing and packaging operations. For example, incorporation into active packaging materials or edible coatings demands stability under industrial conditions such as temperature fluctuations, mechanical stress, and extended storage durations [9]. Moreover, encapsulated bioactives should not be considered as standalone preservation strategies but rather as components of hurdle technology frameworks. Their performance may be strongly influenced by modified atmosphere packaging, high-pressure processing, mild heat treatment, chilled storage, and bioprotective cultures, all of which can alter microbial targets, effective antimicrobial concentrations, and release kinetics. In some cases, such combinations may provide synergistic effects and allow lower bioactive doses, thereby reducing potential sensory impacts, although the extent of synergy remains highly system-dependent and requires product-specific validation [148]. Consequently, encapsulated systems validated under simple aerobic refrigerated conditions should be re-evaluated under commercially relevant hurdle combinations before industrial implementation.

Clean-label requirements and regulatory considerations further influence the industrial adoption of encapsulation technologies. Consumers increasingly demand natural and recognizable ingredients, which limits the use of certain encapsulation materials and processing aids. The commercial application of encapsulated bioactives must therefore be evaluated within market-specific regulatory frameworks. In the European Union, relevant legislation includes Regulation (EU) 2015/2283 on Novel Foods, Regulation (EC) No 1333/2008 on Food Additives, and EFSA guidance documents addressing the risk assessment of nanomaterials in food applications. These frameworks are particularly important for nanoscale delivery systems because changes in particle size, exposure profile, or biological interaction may trigger additional safety assessment requirements. In the United States, novel delivery systems may require evaluation under FDA food additive regulations or the Generally Recognized as Safe (GRAS) framework. Consequently, approval pathways for nano-enabled or novel encapsulation systems may be substantially longer and more complex than those for conventional food-grade carriers, creating practical barriers to commercialization [149].

Consumer acceptance remains a critical factor in the industrial adoption of encapsulated bioactives. Although encapsulation can improve preservation performance by stabilizing natural bioactives and regulating their release, sensory changes associated with bioactive incorporation may still negatively affect product acceptance. This is particularly relevant for essential oils and plant extracts, which may impart strong aroma, flavor, or color attributes if release behavior and dosage are not properly optimized. Moreover, encapsulation should be regarded as an enabling tool for clean-label-oriented preservation rather than as a clean-label attribute in itself. The clean-label suitability of an encapsulated system ultimately depends on the combined effects of carrier composition, processing aids, regulatory acceptance, labeling requirements, and consumer perception. Therefore, successful application requires not only technological efficacy but also sensory optimization and transparent formulation design.

Economic factors also play a critical role in determining feasibility. The cost of encapsulation materials, processing technologies, and additional formulation steps can significantly increase production expenses. Therefore, cost–benefit balance remains a key consideration, particularly for large-scale meat production systems where margins are relatively narrow [147]. Spray drying currently remains the most attractive option for industrial implementation due to its combination of low cost, high throughput, and established regulatory acceptance. Coacervation provides superior protection and controlled release but is more complex to implement at industrial scale. Nanoemulsions and nanoliposomes offer enhanced functionality and improved delivery performance; however, regulatory scrutiny and consumer perception may limit their widespread adoption. Cyclodextrin-based systems may be particularly attractive for clean-label-oriented applications because of their ability to stabilize volatile compounds while minimizing sensory impacts. Therefore, technology selection should balance functional performance with economic feasibility, regulatory requirements, and market positioning.

Beyond technological and economic considerations, the long-term microbiological implications of controlled-release systems remain insufficiently explored. Encapsulation technologies are designed to prolong antimicrobial activity during storage; however, extended exposure of microorganisms to sub-inhibitory concentrations may influence microbial adaptation and reduce preservation efficacy under certain conditions. In controlled-release systems, sub-MIC exposure may occur when the concentration of the released antimicrobial falls below the minimum inhibitory concentration while surviving microbial populations remain exposed for prolonged periods. This issue may be particularly relevant for bacteriocin- and essential-oil-based delivery systems intended for prolonged storage, as sustained release may increase the likelihood of prolonged sub-inhibitory exposure. Although direct evidence linking encapsulated antimicrobial delivery systems to resistance development remains limited, future studies should evaluate release kinetics in relation to MIC thresholds and investigate the microbiological consequences of prolonged sub-MIC exposure under realistic storage conditions. Accordingly, the potential for microbial adaptation should be evaluated alongside antimicrobial efficacy whenever sustained-release systems are proposed for food safety-critical applications.

Future developments are expected to focus on the design of more efficient and adaptable delivery systems. Emerging approaches include stimuli-responsive systems, multi-bioactive formulations with synergistic effects, and hybrid encapsulation strategies that combine multiple technologies to enhance performance. In addition, the integration of artificial intelligence and data-driven tools is gaining attention for predictive modelling of encapsulation efficiency, optimization of release kinetics, selection of carrier and wall materials, and formulation design [150]. These approaches may accelerate formulation development and enable the design of delivery systems tailored to specific meat products and storage conditions.

Advances in sustainable materials and circular-economy approaches are also expected to support the development of eco-friendly encapsulation systems. The use of agro-industrial by-products as sources of bioactive compounds and encapsulation materials represents a promising direction for both functional improvement and environmental sustainability [120]. However, practical applications may be limited by compositional variability associated with raw material origin, seasonal changes, and processing conditions, which can affect encapsulation efficiency, stability, and reproducibility [151]. Improved raw material characterization, process standardization, and quality-control strategies will therefore be essential to ensure consistent performance in large-scale applications.

Overall, bridging the gap between laboratory innovation and industrial application requires multidisciplinary efforts focusing on scalability, regulatory compliance, cost optimization, process standardization, and consumer acceptance. Future progress will depend on developing encapsulation systems that meet functional, economic, regulatory, and clean-label expectations.

## 9. Conclusions

Encapsulation of natural bioactive compounds represents a promising strategy for advancing clean-label preservation in meat systems by improving stability, controlling/modulating release behavior, and enhancing functional performance. However, the effectiveness of these systems is not determined solely by encapsulation efficiency, but by the complex interplay between structural design, release kinetics, and interactions with the meat matrix. Among the available approaches, microencapsulation and emulsion-based systems currently appear to offer the greatest practical feasibility for meat processing applications, owing to their relatively mature production technologies, scalability, compatibility with existing food-processing operations, and ability to improve the stability and controlled release of sensitive bioactives. In contrast, nanoencapsulation and electrospun systems provide higher functional precision and improved release control, but their industrial application may remain more limited in the short term because of regulatory, cost, and scale-up considerations.

Future research should shift from system-centered approaches toward application-driven design strategies, in which encapsulation systems are designed for specific product characteristics, processing conditions, and storage environments. In particular, integrating realistic food models, optimizing release profiles, and ensuring sensory compatibility will be essential for successful implementation. Future studies should prioritize the validation of encapsulated bioactives in real meat products under industrially relevant storage and packaging conditions, the optimization of dose–release–sensory acceptability relationships, and the assessment of regulatory and clean-label compatibility of carrier materials. In the longer term, fundamental studies are needed to clarify how meat matrix composition, spoilage dynamics, and controlled-release kinetics interact to determine preservation efficacy.

Bridging the gap between laboratory-scale innovation and industrial application requires the development of scalable, cost-effective, and regulatory-compliant systems. Ultimately, the rational design of encapsulation systems based on structure–function relationships and system-level compatibility will be key to the practical and sustainable use of natural bioactives in next-generation meat preservation strategies.

## Figures and Tables

**Table 1:** Encapsulation strategies for natural bioactives in meat systems.

Challenge in Meat Systems	Impact on Bioactives	Role of Encapsulation	Example of Application	Main Limitations	Reference
Chemical instability (oxidation, degradation)	Oxidative reactions in the meat matrix reduce antioxidant activity of bioactives during storage	Encapsulation shields bioactives from oxygen and reactive species, preserving activity	Grape pomace phenolics encapsulated in maltodextrin reduced TBARS in beef burgers during storage	Possible incomplete protection during long storage; carrier selection is critical	[71]
Volatility and aroma loss (essential oils)	Rapid evaporation of volatile compounds reduces antimicrobial efficacy and causes sensory imbalance	Capsule walls act as diffusion barriers, enabling controlled release and retention of volatiles	Spray-dried thyme essential oil retained antimicrobial activity in hamburger systems	Release may be too slow or insufficient for immediate antimicrobial action	[72]
Poor water solubility and dispersion	Hydrophobic bioactives reveal limited distribution and reduced interaction with microbial and oxidative targets	Emulsification and nanoparticle systems improve solubility and dispersion in meat matrices	Curcumin-loaded chitosan nanoparticles enhanced solubility and reduced oxidation in pork systems	Physical instability, aggregation, or phase separation may occur	[73]
Strong sensory impact (off-flavors, bitterness)	Direct addition leads to undesirable flavor and reduced consumer acceptance	Encapsulation masks flavor and delays release, reducing sensory perception	Gallic acid microcapsules improved oxidative stability without affecting sensory quality in hamburgers	Flavor masking may be incomplete at high bioactive concentrations	[74]
Interactions with meat components (proteins, lipids)	Binding to proteins and lipids reduces bioavailability and alters meat structure	Encapsulation prevents premature interactions and enables controlled release	Encapsulated catechin maintained texture and reduced oxidation in beef meatballs	Encapsulation may alter texture or release behavior depending on the matrix	[75]
Rapid release and short-lived activity	Bioactives lose effectiveness quickly due to uncontrolled release and degradation	Controlled and sustained release systems maintain effective concentrations over time	Cyclodextrin-based systems prolonged antimicrobial activity in pork products	Release kinetics may not match spoilage dynamics during storage	[76]
Sensitivity to processing conditions (heat, shear)	Thermal and mechanical treatments degrade sensitive compounds	Encapsulation improves resistance to heat and processing stresses	Alginate-protein encapsulated fish oil retained stability after heat treatment in meat systems	Thermal protection depends strongly on wall material and process conditions	[77]
Scale-up and stability limitations	Some systems lack industrial feasibility and stability under processing conditions	Use of scalable materials and techniques improves industrial applicability	Nanoemulsion systems improved vitamin D stability in meat matrices under processing conditions	Scale-up, cost, regulatory approval, and long-term stability remain challenging	[78]

**Table 2:** Selected studies on encapsulated natural bioactives applied in meat products.

Source of Bioactive Compounds	Encapsulation System	Meat Product	Storage Conditions	Key Findings	Reference
Ginger essential oil; Thyme essential oil; Lemongrass essential oil	Nanoemulsion	Minced beef	4 °C, 18 days	Improved sensory and microbial quality; lemongrass nanoemulsion extended shelf life by approximately 6 days compared with control samples.	[133]
Orange essential oil + xoconostle	W/O nanoemulsion	Emulsified meat system	4 °C, 60 days	Increased antioxidant activity; reduced lipid oxidation by approximately 2.7-fold.	[134]
Carvacrol; Cinnamaldehyde; Citral; Eugenol	Nanoemulsion coating	Pork tenderloin	4 °C, 9 days	Reduced oxidation and microbial growth; microbial counts decreased by up to ~2–3 log CFU/g during refrigerated storage.	[135]
Cinnamon essential oil + Turmeric essential oil	Nanoemulsion	Minced beef	Room temp., 24 h	Reduced TBARS and metmyoglobin; TBARS values decreased by approximately 30–40% compared with control.	[136]
Thyme essential oil; Oregano essential oil	Chitosan nanoparticles	Meat emulsion	4 °C, 15 days	Lower TBARS values and delayed microbial growth during 15-day refrigerated storage.	[137]
Cinnamon essential oil; Ginger essential oil	Solid lipid nanoparticles	Beef burger	−18 °C, 6 months	Reduced TBARS; enhanced antioxidant activity; shelf life extended by approximately 2-fold compared with free essential oil.	[93]
Rosemary extract	Nanoencapsulation	Beef fillets	4 °C, 28 days	Extended shelf life from approximately 14 to 28 days under refrigerated storage.	[138]
Green tea extract + flaxseed oil	Chitosan coating	Beef cuts	4 °C, 8 days	Lower TBARS formation and improved redness (a*) retention during storage.	[139]
Prunus domestica (anthocyanin-rich) extract + epigallocatechin gallate	Electrospinning	Fresh beef meat	4 °C, 15 days	Delayed spoilage-related quality changes and improved freshness indication during refrigerated storage.	[140]
Nisin + avocado peel extract	Microencapsulation	Ground beef	4 °C, 10 days	Reduced bacterial growth and protein oxidation; extended shelf life; microbial counts reduced by approximately 2 log CFU/g.	[141]
Hibiscus extract (phenolic-rich)	Nanoencapsulation (CMC-based)	Chicken nuggets	4 °C, 9 days	Lower oxidation levels and higher sensory acceptability compared with control samples.	[44]
Boletus edulis mushroom extract (polyphenols)	Microencapsulation	Vienna sausages	4 °C, 8 days	Higher antioxidant capacity and reduced oxidative deterioration during storage.	[142]
Pitaya peel extract	Spray drying (maltodextrin)	Pork patties	4 °C, 30 days	Delayed protein oxidation and improved stability; protein carbonyl formation significantly reduced during 30-day storage.	[143]

## Data Availability

No new data were created or analyzed in this study. Data sharing is not applicable to this article.

## Supplementary Materials

The following supporting information can be downloaded at: https://www.mdpi.com/article/10.3390/foods15132407/s1, Figure S1: PRISMA 2020 flow diagram summarizing the literature search and study selection process.
