# Supplementary figures and images for: Encapsulation Strategies for Natural Bioactives in Clean-Label Meat Preservation: A Review
Source: Foods. 2026 Jul 7;15(13):2407. doi: 10.3390/foods15132407 (PMC13361067; doi:10.3390/foods15132407)

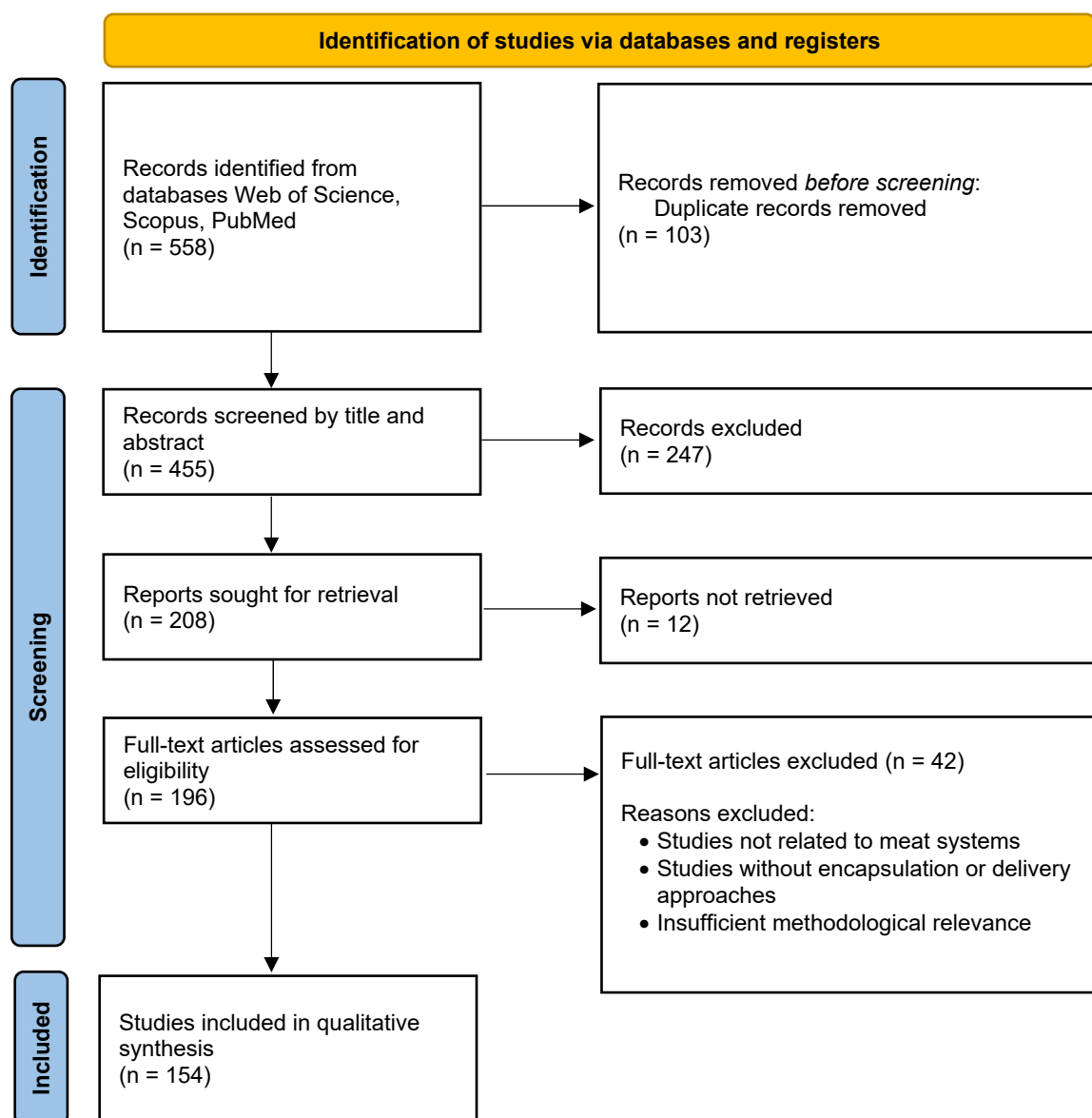

**Figure S1.** PRISMA 2020 flow diagram summarizing the literature search and study selection process.

Supplement: Supplementary file 1 [file foods-15-02407-s001.zip › foods-4317253-supplementary.pdf]
